# Polymer-Functionalized Mitochondrial Transplantation to Fibroblasts Counteracts a Pro-Fibrotic Phenotype

**DOI:** 10.3390/ijms241310913

**Published:** 2023-06-30

**Authors:** Gherardo Baudo, Suhong Wu, Matteo Massaro, Haoran Liu, Hyunho Lee, Aijun Zhang, Dale J. Hamilton, Elvin Blanco

**Affiliations:** 1 Department of Nanomedicine, Houston Methodist Research Institute, Houston, TX 77030, USA; 2 College of Materials Sciences and Opto-Electronic Technology, University of Chinese Academy of Sciences, Beijing 100049, China; 3 Center for Bioenergetics, Houston Methodist Research Institute, Houston, TX 77030, USA; 4 Department of Medicine, Weill Cornell Medical College, New York, NY 10065, USA; 5 Division of Endocrinology, Diabetes, and Metabolism, Department of Medicine, Houston Methodist Hospital, Houston, TX 77030, USA; 6 Department of Cardiology, Houston Methodist DeBakey Heart and Vascular Center, Houston Methodist Hospital, Houston, TX 77030, USA

**Keywords:** mitochondrial transplantation, fibroblasts, transforming growth factor-β, fibroblast-to-myofibroblast transition, glycolysis

## Abstract

Fibroblast-to-myofibroblast transition (FMT) leads to excessive extracellular matrix (ECM) deposition—a well-known hallmark of fibrotic disease. Transforming growth factor-β (TGF-β) is the primary cytokine driving FMT, and this phenotypic conversion is associated with mitochondrial dysfunction, notably a metabolic reprogramming towards enhanced glycolysis. The objective of this study was to examine whether the establishment of favorable metabolic phenotypes in TGF-β-stimulated fibroblasts could attenuate FMT. The hypothesis was that mitochondrial replenishment of TGF-β-stimulated fibroblasts would counteract a shift towards glycolytic metabolism, consequently offsetting pro-fibrotic processes. Isolated mitochondria, functionalized with a dextran and triphenylphosphonium (TPP) (Dex-TPP) polymer conjugate, were administered to fibroblasts (MRC-5 cells) stimulated with TGF-β, and effects on bioenergetics and fibrotic programming were subsequently examined. Results demonstrate that TGF-β stimulation of fibroblasts led to FMT, which was associated with enhanced glycolysis. Dex-TPP-coated mitochondria (Dex-TPP/Mt) delivery to TGF-β-stimulated fibroblasts abrogated a metabolic shift towards glycolysis and led to a reduction in reactive oxygen species (ROS) generation. Importantly, TGF-β-stimulated fibroblasts treated with Dex-TPP/Mt had lessened expression of FMT markers and ECM proteins, as well as reduced migration and proliferation. Findings highlight the potential of mitochondrial transfer, as well as other strategies involving functional reinforcement of mitochondria, as viable therapeutic modalities in fibrosis.

## 1. Introduction

Fibrosis represents the ultimate pathological outcome of a dysregulated wound healing response following severe or repetitive tissue injury that is present in several chronic inflammatory diseases [1]. One example includes idiopathic pulmonary fibrosis (IPF), which arises from persistent injury to the pulmonary epithelium followed by an aberrantly activated tissue repair response [2]. Similarly, an exaggerated immune response and injury to hepatocytes are observed in nonalcoholic steatohepatitis (NASH), the severe form of nonalcoholic fatty liver disease (NAFLD), ultimately resulting in the deposition of scar tissue in the liver [3]. Lastly, cardiac fibrosis stems from stresses such as chronic high blood pressure and ischemic injury [4], with persistent and repetitive injury resulting in continual deposition of fibrous tissue [5]. Ultimately, fibrosis leads to organ failure in these and other diseases (e.g., kidney disease), as parenchymal tissue is replaced and disrupted by a disproportionate amount of extracellular matrix (ECM) deposition [6].

All pathways, cytokines, and growth factors in the pathogenesis of fibrosis ultimately converge at the activation of fibroblasts, the principal ECM producing cell and effector whose activation, proliferation, differentiation into myofibroblasts, and survival are key processes involved in fibrotic progression [7]. Transforming growth factor-β (TGF-β) is the key activator of fibroblasts and a driver of fibroblast-to-myofibroblast transition (FMT) through increased Smad-dependent signaling [8], eventually resulting in the pervasive hallmark of fibrosis that is excessive accumulation of ECM components such as collagen [9]. TGF-β signaling is also a central regulator of a broad range of cellular processes, including proliferation, migration, gene expression, apoptosis, and adhesion [10]. The significant role of TGF-β as a mediator of fibrosis is evidenced by its persistent induction and activation in patients with fibrotic disorders and its ability to be used as a predictor of the progression and severity of fibrosis [8]. 

Aberrant TGF-β signaling [11,12] and fibroblast activation [13,14,15] are associated with mitochondrial dysfunction. TGF-β increases aerobic glycolysis during fibroblast differentiation, and hypoxia-inducible factor-1α (HIF-1α) is upregulated [14,16,17]. Glutaminolysis, increased glycolytic flux, and decreased glucose oxidation provide biomass for cell proliferation, growth, and differentiation and supply the energy demand required by the myofibroblast contractile phenotype [18]. Glycolysis generates biosynthetic intermediates that support the production and secretion of ECM components such as collagen. Mitochondrial impairments stemming from defects in the electron transport chain (ETC) [19] and mitochondrial fission imbalances [20], to name a few, lead to mitochondrial-derived ROS (mtROS), which in turn proves key for fibroblast differentiation. Given the important role that mitochondrial dysfunction plays in FMT, targeting metabolic and mitochondrial abnormalities in fibroblasts represents a viable treatment option in a variety of fibrotic disease states.

**Figure 1:**
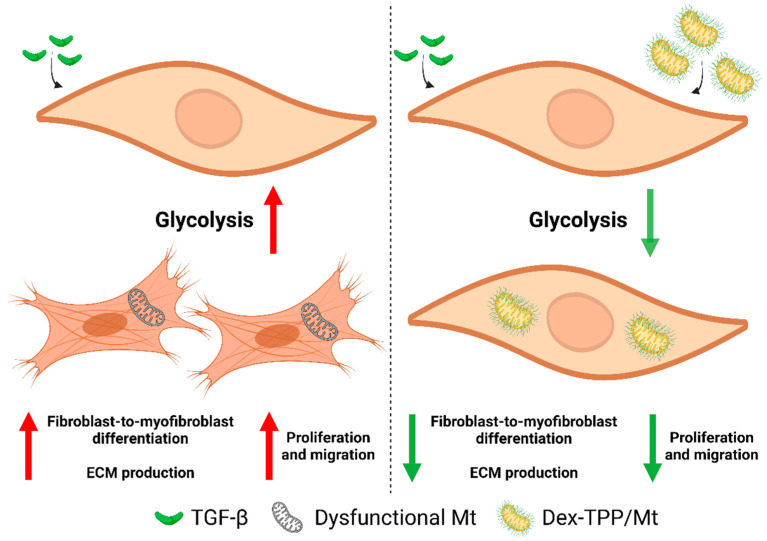
Dex-TPP/Mt treatment of TGF-β-stimulated fibroblasts. TGF-β stimulation of fibroblasts is associated with an increase in glycolysis and pro-fibrotic processes, including myofibroblast conversion, ECM production, and cell proliferation and migration. Transfer of Dex-TPP/Mt to TGF-β-stimulated fibroblasts was hypothesized to deter glycolytic programming and offset pro-fibrotic cell dynamics.

We formulated a dextran (Dex) and triphenylphosphonium (TPP) polymer conjugate (Dex-TPP) for functionalization of isolated mitochondria (Dex-TPP/Mt) [21]. Dextran was chosen due to its wide use in biomedical applications as well as its non-toxicity, biocompatibility, and biodegradability [22]. TPP, a lipophilic cation, was selected based on its mitochondriotropic nature [23], which allowed for stable association of Dex with mitochondrial membranes. The resulting polymer-coated organelles were previously shown to enhance cellular transplantation. Dex-TPP/Mt had a ~3-fold higher internalization in various cell lines compared to uncoated mitochondria, and metabolically reprogrammed breast cancer cells and cardiomyocytes away from glycolysis [21]. Additionally, theranostic advantages of Dex-TPP functionalization of mitochondria exist, including the ability to incorporate imaging probes (e.g., fluorescent dyes) for prompt identification of tissue localization and persistence, as well as further functionalization with capabilities for enhanced cell-specific targeting and internalization. Dex-TPP can also serve as therapeutic carriers through the incorporation of agents that bolster synergistic effects. Recently, we demonstrated that Dex-TPP/Mt transplantation reduced glycolysis in classically activated macrophages and decreased pro-inflammatory cytokine production [24]. The objective of this study was to induce favorable metabolic profiles in activated fibroblasts. We hypothesized that delivery of Dex-TPP/Mt to TGF-β-stimulated fibroblasts would offset aberrant mitochondrial bioenergetics and attenuate their pro-fibrotic phenotype (Figure 1). Our results show that TGF-β increased the expression of FMT markers and that this increase was associated with enhanced glycolysis. Dex-TPP/Mt treatment of TGF-β-stimulated fibroblasts attenuated a metabolic shift towards glycolysis and resulted in lessened reactive oxygen species (ROS) generation. TGF-β-stimulated fibroblasts treated with Dex-TPP/Mt had reduced expression of FMT markers and ECM proteins. Dex-TPP/Mt treatment also reduced myofibroblast migration and proliferation. Findings highlight the potential of mitochondrial transfer as a novel therapeutic for fibrotic diseases and underline the viability of fibrosis treatment strategies aimed at restoring proper bioenergetics and mitochondrial dynamics.

## 2. Results and Discussion

### 2.1. TGF-β Stimulation of Fibroblasts Promoted Myofibroblast Differentiation and a Pro-Fibrogenic Programming

**Figure 2:**
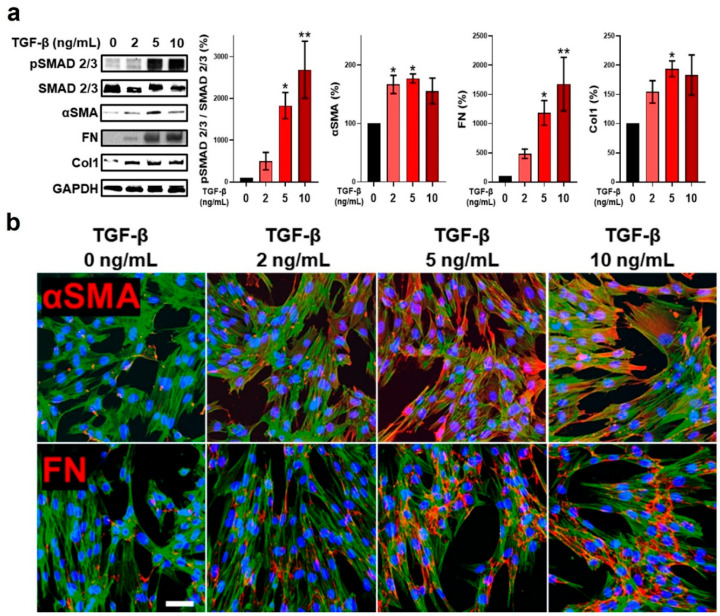
Effect of TGF-β on fibroblast pro-fibrotic processes. (**a**) Representative Western blot of Smad signaling, myofibroblast marker, and ECM protein expression, as well as relative protein level quantification, in fibroblasts following TGF-β stimulation for 24 h. Protein markers were normalized to GAPDH expression levels (n = 3). (**b**) Representative immunofluorescence micrographs highlighting expression of αSMA and FN in fibroblasts following TGF-β stimulation for 24 h. αSMA or FN appear red, F-actin is represented in green, and DAPI-stained nuclei appear blue. The scale bar represents 100 µm. * *p* < 0.05; ** *p* < 0.01 vs. 0 ng/mL TGF-β group.

The overexpression and persistent induction of TGF-β play a pivotal role in fibrosis in a variety of disease states and organs [25]. In hepatic fibrosis, TGF-β results in an increase in ECM proteins and collagen synthesis [26,27]. In IPF, TGF-β promotes a fibroblast-to-myofibroblast phenotypic transition as well as collagen and filamentous actin production [28]. Lastly, in infarcted hearts, increased TGF-β mRNA levels are associated with increased mRNA levels of ECM proteins [29]. TGF-β exerts a direct, central effect on fibroblasts through canonical signaling involving the Smad family of proteins [30]. While all three TGF-β isoforms are involved in Smad signaling-dependent fibrosis, TGF-β1 results in a more severe and pronounced fibrotic response compared to the other isoforms of the TGF-β family [31]. Herein, fibroblast stimulation with increasing concentrations of TGF-β1 resulted in increased Smad2/3 phosphorylation (Figure 2a), a finding indicative of Smad2/3 signaling pathway activation [32]. Upon phosphorylation, Smad2/3 forms complexes with Smad4 and undergoes nuclear translocation for transcriptional regulation of target genes implicated in fibrosis progression, including those involved in ECM deposition and fibroblast migration and proliferation [33]. Fibroblasts stimulated with TGF-β1 had increased proliferation compared to naïve fibroblasts (Supplementary Figure S1). Notably, a concentration of 10 ng/mL had detrimental effects on cell viability, as determined via MTT assay (Supplementary Figure S2). Smad signaling is also an important pathway regulating the expression of α-smooth muscle actin (αSMA) [34], an important marker of myofibroblast conversion, and, along with actin-myosin bundles, one of the distinguishing characteristics of these cells [35,36]. Fibroblast phenotypic conversion to myofibroblasts represents the crucial step that drives ECM remodeling and maintenance [37], with FMT proving a prerequisite for increased collagen production [38]. Stimulation of fibroblasts with TGF-β1 resulted in enhanced αSMA expression, as evidenced by analysis of protein expression (Figure 2a) and immunofluorescence (Figure 2b and Supplementary Figure S3). Myofibroblasts exhibit enhanced rates of assembly of the ECM adhesive protein fibronectin (FN) compared to non-differentiated fibroblasts [39], and αSMA-expressing myofibroblasts represent the predominant source of type I collagen (Col1) [40]. TGF-β1 stimulation of fibroblasts led to an increase in FN expression (Figure 2a,b and Supplementary Figure S4), as well as the ECM structural protein Col1 (Figure 2a). Assembly of a FN fibrillar matrix is essential for new ECM formation, serving as a scaffold for collagen binding and other ECM proteins [41], and as a repository for varied growth factors, such as vascular endothelial growth factor (VEGF) and fibroblast growth factor 2 (FGF-2), that play important roles in fibrosis and tissue repair [42]. Col1 is known to stimulate TGF-β upregulation and a pro-fibrotic phenotype in fibroblasts [43]. Notably, αSMA and Col1 expression did not demonstrate a further increase at a TGF-β1 dose of 10 ng/mL. This dose of TGF-β1 has previously been shown to inhibit cell proliferation and even induce apoptosis [44], mirroring our own findings in this study (Supplementary Figure S2). High concentrations of TGF-β can activate stress-related signaling pathways [45,46,47] that may result in cell death and prevent the expression of genes necessary for cell growth and differentiation [48]. Tang et al. reported that TGF-β at concentrations ≥10 ng/mL resulted in increased GAS5 expression due to a feedback response to inhibition of cell proliferation [49]. GAS5 overexpression, in turn, suppressed both αSMA and Col1A protein expression. Our results involving αSMA and Col1 expression at a concentration of 10 ng/mL agree with other previously published reports. Chen et al. demonstrated that αSMA reached maximal expression levels in human vocal fold fibroblasts (hVFF) treated with 5 and 10 ng/mL of TGF-β1, with a slight decrease observed at 10 ng/mL [50]. Fragiadaki et al. demonstrated that CUX1, a CCAAT displacement protein, suppressed type I collagen at a 10 ng/mL dose of TGF-β [45].

### 2.2. TGF-β Stimulation of Fibroblasts Led to Metabolic Reprogramming of Fibroblasts towards Glycolysis

**Figure 3:**
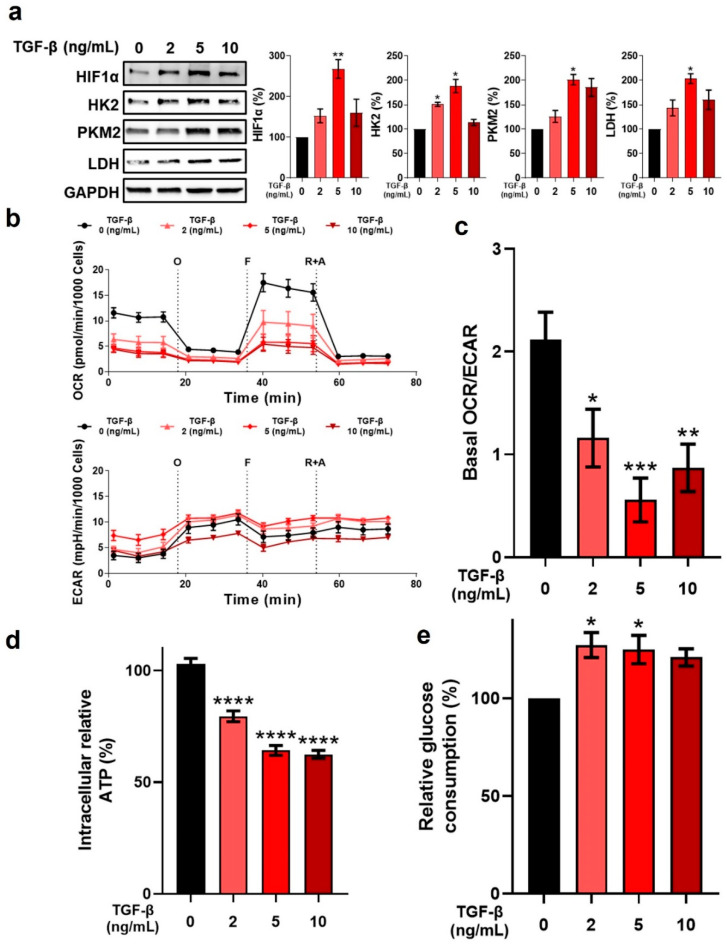
Effect of TGF-β on fibroblast metabolic phenotype and bioenergetics. (**a**) Representative Western blot of HIF-1α and glycolytic enzyme expression, as well as relative protein level quantification, in fibroblasts following TGF-β stimulation for 24 h. Protein markers were normalized to GAPDH expression levels (n = 3). (**b**) Bioenergetic examination of the effect of TGF-β on fibroblasts after 24 h. Upper panel: oxygen consumption rate (OCR) in fibroblasts (n = 3). Lower panel: extracellular acidification rate (ECAR) in fibroblasts (n = 3). O: oligomycin; F: FCCP; R + A: rotenone + antimycin A. (**c**) The ratio of basal OCR/ECAR of fibroblasts. (**d**) Relative intracellular ATP (n = 3). (**e**) Relative glucose consumption (n = 3). * *p* < 0.05; ** *p* < 0.01; *** *p* < 0.001; **** *p* < 0.0001 vs. 0 ng/mL TGF-β group.

TGF-β stimulation of fibroblasts is associated with accentuated metabolic activity [8]. Si et al. recently demonstrated that TGF-β stimulates hyperglycolysis, resulting in an upregulation of glycolytic enzymes [51]. HIF-1α is an important mediator of glycolytic enzyme activity, and its increased expression has previously been observed in myofibroblasts compared to quiescent fibroblasts [16,17]. Notably, sustained HIF-1α exposure has been associated with persistent pathofibrogenesis [52]. Herein, HIF-1α expression in fibroblasts was upregulated following TGF-β1 stimulation (Figure 3a). Notably, the mechanism by which TGF-β increases expression of HIF-1α, and that of several glycolytic enzymes remains elusive. McMahon et al. demonstrated that TGF-β decreased the expression of prolyl hydroxylase domain-containing protein 2 (PHD2), an enzyme associated with HIF-1α degradation [53]. Basu et al., showed a signaling cooperation between HIF-1α and Smad3, wherein blocking of Smad3 activity resulted in inhibition of various HIF-1α-mediated activities, with findings suggesting that a TGF-β1-mediated increase in HIF-1α expression is due to increased HIF-1α mRNA translation into protein [54]. In lung myofibroblasts, enhanced glycolysis and a consequent increase in the levels of succinate were found to stabilize HIF-1α [14]. An enhanced glycolytic rate contributes to the production of biomass needed for fibroblast proliferation and differentiation, the energy requirements of myofibroblasts, and biosynthetic intermediates for the production and secretion of ECM components such as collagens [18]. TGF-β1 stimulation of fibroblasts increased the expression of key glycolytic enzymes, including hexokinase-2 (HK2, Figure 3a). Hexokinase was recently identified as one of the four key glycolytic flux-controlling steps [55], and HK2 is an important enzyme for the phosphorylation of glucose [56]. Yin et al. demonstrated that a TGF-β-induced activation of Smad2/3 resulted in increased c-Myc signaling, which in turn resulted in increased HK2 expression in fibroblasts [57]. In the same study, HK2 proved to be an important mediator of the fibroproliferative activity of TGF-β, including the stimulation of myofibroblast conversion, induction of ECM gene synthesis, and migration [57]. Expression of pyruvate kinase isozyme M2 (PKM2) was also found to be upregulated in fibroblasts following TGF-β1 stimulation (Figure 3a). PKM2 is an enzyme that catalyzes the conversion of phosphoenolpyruvate (PEP) to pyruvate [58]. Increased c-Myc was also shown to result in alternative splicing of PKM to PKM2, which in turn contributed to a metabolic reprogramming towards glycolysis [59]. Gao et al. recently reported that PKM2 promoted progression of fibrosis through direct interaction with Smad7 and a reinforcement of TGF-β signaling [60]. Lastly, lactate dehydrogenase (LDH) expression was increased in fibroblasts following TGF-β1 stimulation (Figure 3a). Kottman et al. demonstrated that TGF-β-mediated induction of HIF-1α expression led to an increase in LDH5 expression, with heightened LDH5 in fibroblasts leading to lactic acid production [61]. Moreover, findings from the same study suggest the existence of a potential feed-forward loop wherein lactic acid activates latent TGF-β, which in turn increases HIF-1α and LDH5 expression. While LDH is responsible for lactate production and transition to anaerobic glycolysis, with increased lactate having been observed in myofibroblasts [16,61,62], its role in myofibroblast conversion induction remains unclear [61,63].

TGF-β1 stimulation had profound effects on fibroblast bioenergetics. Analysis of basal OCR demonstrated that TGF-β1 stimulation of fibroblasts caused a pronounced decrease in basal respiration in a dose-dependent manner compared to naïve fibroblasts (Figure 3b). 

Similar results were observed for maximal respiratory capacity (Figure 3b). ECAR analysis after TGF-β1 stimulation showed an increase in basal ECAR compared to naïve fibroblasts (Figure 3b), signaling an increase in glycolysis. The basal OCR/ECAR ratio, an indication of cell bioenergetic balance and a comparator of how oxidative pathways and glycolysis are used for energy production, was significantly decreased following TGF-β1 stimulation of fibroblasts (Figure 3c). Of note, TGF-β1 stimulation of fibroblasts led to a significant decrease in intracellular ATP compared to naïve fibroblasts (Figure 3d) and an increase in relative glucose consumption (Figure 3e), the latter a reflection of the slightly elevated glycolytic activity of these cells.

### 2.3. Dex-TPP/Mt Underwent Uptake by TGF-β-Stimulated Fibroblasts

To metabolically reprogram TGF-β-stimulated fibroblasts away from glycolysis and hinder a pro-fibrotic phenotype, we examined a strategy involving the delivery of healthy mitochondria functionalized with a Dex-TPP conjugate to fibroblasts. Previously, our laboratory demonstrated that incorporation of Dex-TPP into mitochondria resulted in efficient uptake in a variety of cells compared to uncoated mitochondria [21]. Importantly, and in agreement with previous results from our group [21,24], Dex-TPP/Mt had no detrimental effects on fibroblast viability. Herein, Dex-TPP/Mt was internalized by TGF-β1-stimulated fibroblasts, agreeing with previous cell uptake results from our laboratory [21,24]. At a timepoint of 24 h, flow cytometry assays confirmed the presence of Dex-TPP/Mt in fibroblasts (Supplementary Figure S5a), with confocal microscopy analysis corroborating Dex-TPP/Mt uptake (Supplementary Figure S5b).

### 2.4. Dex-TPP/Mt Altered the Metabolic Phenotype of TGF-β-Stimulated Fibroblasts

**Figure 4:**
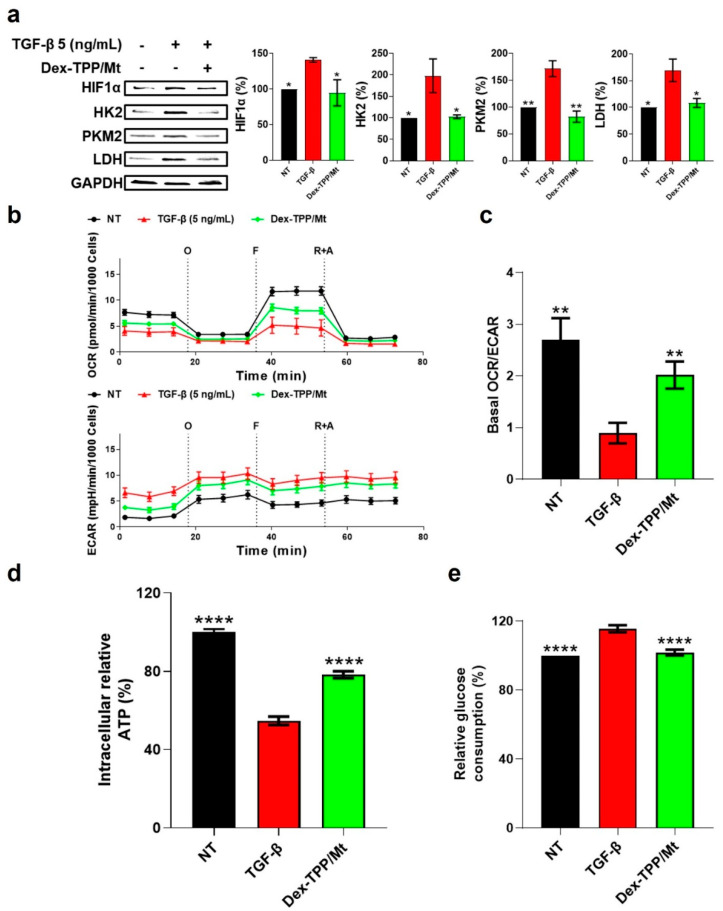
Effect of Dex-TPP/Mt treatment on metabolic phenotype and bioenergetics in TGF-β-stimulated fibroblasts. (**a**) Representative Western blot of HIF-1α and glycolytic enzyme expression, as well as relative protein level quantification, in non-treated fibroblasts (NT), TGF-β-stimulated fibroblasts (TGF-β), and TGF-β-stimulated fibroblasts treated with Dex-TPP/Mt for 24 h. Protein markers were normalized to GAPDH expression levels (n = 3). (**b**) Bioenergetic examination of the effect of Dex-TPP/Mt treatment on TGF-β-stimulated fibroblasts for 24 h. Upper panel: oxygen consumption rate (OCR) in fibroblasts (n = 3). Lower panel: extracellular acidification rate (ECAR) in fibroblasts (n = 3). O: oligomycin; F: FCCP; R + A: rotenone + antimycin A. (**c**) The ratio of basal OCR/ECAR of fibroblasts. (**d**) Relative intracellular ATP (n = 3). (**e**) Relative glucose consumption (n = 3). * *p* < 0.05; ** *p* < 0.01; **** *p* < 0.0001 vs. TGF-β group.

We aimed to determine whether Dex-TPP/Mt could impact the glycolytic phenotype of TGF-β-stimulated fibroblasts. Dex-TPP/Mt treatment of fibroblasts stimulated with TGF-β1 resulted in a decrease in HIF-1α expression (Figure 4a). Dex-TPP/Mt transplantation also significantly reduced the expression of the glycolytic enzymes HK2, PKM2, and LDH (Figure 4a). Dex-TPP/Mt treatment had profound effects on cell respiration by increasing both basal and maximal OCR, as well as decreasing basal ECAR, in fibroblasts stimulated with TGF-β1 (Figure 4b). Consequently, the OCR/ECAR ratio increased TGF-β1-stimulated fibroblasts treated with Dex-TPP/Mt (Figure 4c). Notably, transplantation of Dex-TPP/Mt resulted in a significant increase in relative intracellular ATP in TGF-β1-stimulated fibroblasts (Figure 4d), indicative of an increase in oxidative phosphorylation (OXPHOS) and cell respiration [64], while glucose consumption significantly decreased as well (Figure 4e). 

Taken together, our findings highlight that TGF-β1-stimulated fibroblasts treated with Dex-TPP/Mt exhibit a reduced glycolytic phenotype and enhanced mitochondrial respiration. We previously showed that Dex-TPP/Mt increased the basal OCR of triple-negative breast cancer (TNBC) cell lines, H9c2 cardiomyocytes (CMs), and adult mouse CMs [21], as well as classically activated bone marrow-derived macrophages (BMDMs) [24]. These findings regarding bioenergetic alterations also agree with studies involving mitochondrial transplantation from other groups. Ali Pour et al. demonstrated an increase in basal and maximal respiration, as well as ATP production, after transplantation of L6 skeletal cell-derived mitochondria to H9c2 CMs [65]. Increased OCR and ATP production were observed in the tibialis anterior muscle of a mouse model of Duchenne muscular dystrophy following muscle stem cell mitochondrial transplantation [66]. Zhang and coworkers showed increased OCR in U87 human glioma cells following astrocyte-derived mitochondrial transplantation [67]. Recently, Baack and coworkers demonstrated that the transfer of normal rat myocardium-derived mitochondria to pregestational diabetes mellitus (PGDM)-exposed, high-fat (HF) diet-exposed, and a combination of PGDM- and HF-exposed cardiomyocytes resulted in a respiratory boost [68]. Overall, these studies highlight the potential of mitochondrial transfer as a strategy to bioenergetically reprogram cells. 

**Figure 5:**
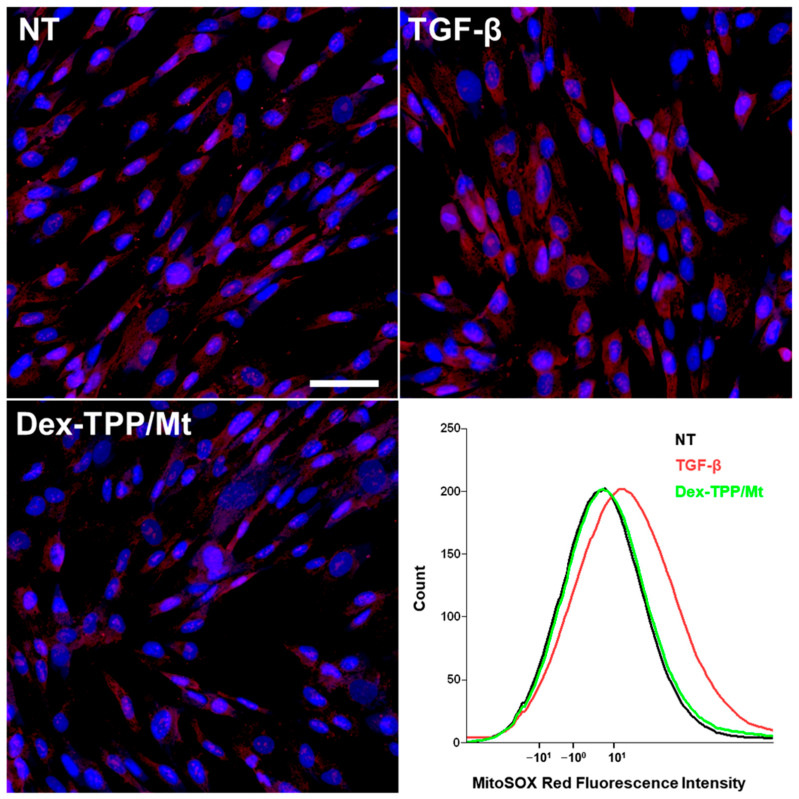
Effect of Dex-TPP/Mt treatment on oxidative stress in TGF-β-stimulated fibroblasts. Representative confocal micrographs of non-treated fibroblasts (NT), TGF-β-stimulated fibroblasts (TGF-β), and TGF-β-stimulated fibroblasts treated with Dex-TPP/Mt for 24 h and subjected to the MitoSOX assay and an accompanying flow cytometry (n = 1). The superoxide anion is represented in red, and DAPI-stained nuclei appear blue. The scale bar represents 100 µm.

TGF-β has been shown to enhance ROS formation principally through NADPH oxidase 4 (NOX4) induction [69], and as mentioned previously, ROS is key for fibroblast differentiation. Herein, TGF-β1 stimulation of fibroblasts resulted in increased ROS production compared to naïve fibroblasts (Figure 5). Dex-TPP/Mt treatment of TGF-β1 stimulated fibroblasts resulted in a reduction in ROS, potentially contributing towards counteracting TGF-β-induced fibroblast conversion. Of note, our findings regarding the effect of mitochondrial transplantation on ROS production agree with those from previous groups [70].

### 2.5. Dex-TPP/Mt Counteracted the Pro-Fibrotic Phenotype and Cell Dynamics of TGF-β-Stimulated Fibroblasts

**Figure 6:**
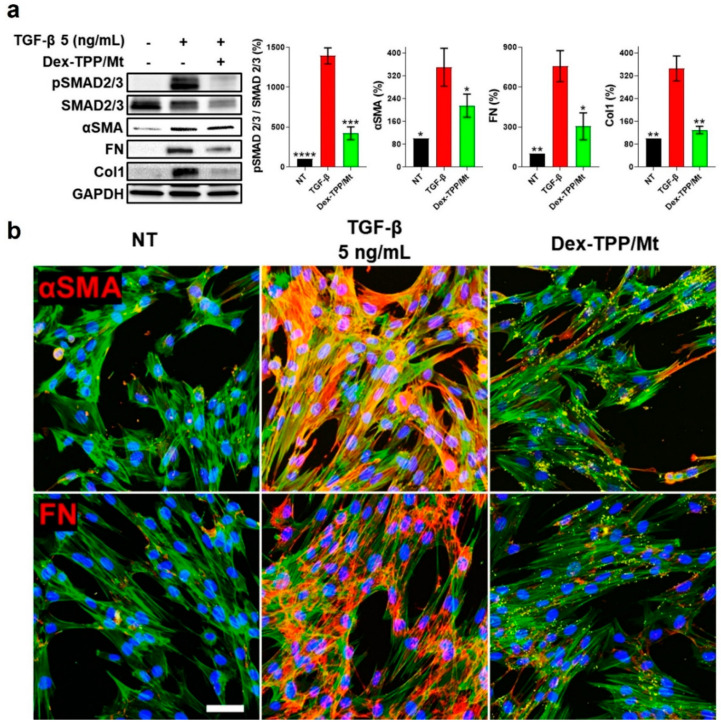
Effect of Dex-TPP/Mt treatment on TGF-β-stimulated fibroblast pro-fibrotic processes. (**a**) Representative Western blot of Smad signaling, myofibroblast marker, and ECM protein expression, as well as relative protein level quantification, in non-treated fibroblasts (NT), TGF-β-stimulated fibroblasts (TGF-β), and TGF-β-stimulated fibroblasts treated with Dex-TPP/Mt for 24 h. Protein markers were normalized to GAPDH expression levels (n = 3). (**b**) Representative immunofluorescence micrographs highlighting expression of αSMA and FN in TGF-β-stimulated fibroblasts following Dex-TPP/Mt treatment labeled with FITC for 24 h. αSMA or FN appear red, F-actin is represented in green, FITC-labeled Dex-TPP/Mt appear yellow, and DAPI-stained nuclei appear blue. The scale bar represents 100 µm. * *p* < 0.05; ** *p* < 0.01; *** *p* < 0.001; **** *p* < 0.0001 vs. TGF-β group.

TGF-β is the archetypal cytokine promoting fibroblast to myofibroblast differentiation, proliferation, and migration, as well as ECM production [43], meriting the examination of the effect of Dex-TPP/Mt on the pro-fibrotic phenotype of TGF-β-stimulated fibroblasts. Following Dex-TPP/Mt transplantation, TGF-β1-induced Smad signaling decreased significantly, as evidenced by downregulated expression of phosphorylated Smad2/3 (Figure 6a). Dex-TPP/Mt treatment also resulted in a significant decrease in the expression of αSMA in TGF-β1-stimulated fibroblasts (Figure 6a,b and Supplementary Figure S6), an effect that also extended to the ECM proteins FN (Figure 6a,b and Supplementary Figure S7) and collagen type I (Figure 6a). Given that a reduction in collagen gene expression is associated with a downregulation of αSMA expression [71], our results point towards the absence of a fully differentiated myofibroblast phenotype following Dex-TPP/Mt treatment. 

**Figure 7:**
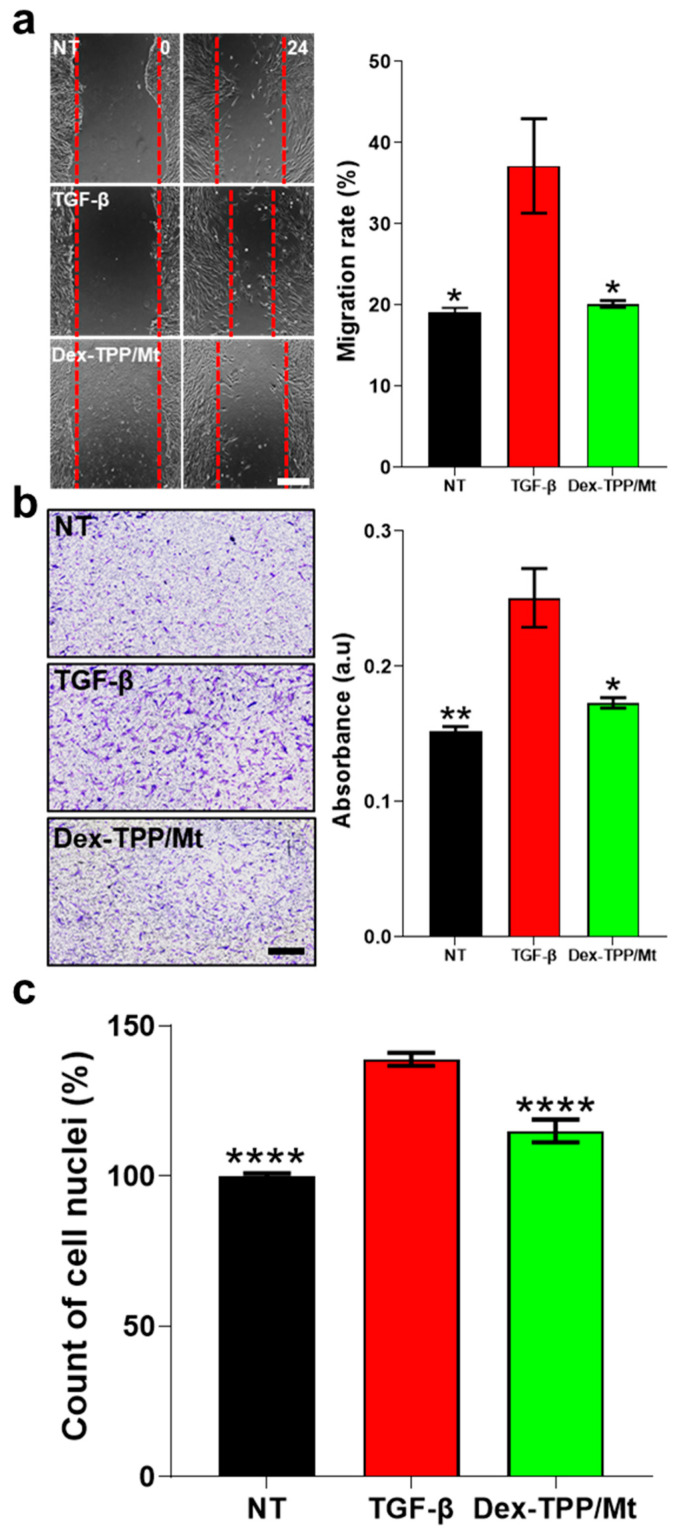
Effect of Dex-TPP/Mt treatment on TGF-β-stimulated fibroblast cell dynamics. Migration of non-treated fibroblasts (NT), TGF-β-stimulated fibroblasts (TGF-β), and TGF-β-stimulated fibroblasts treated with Dex-TPP/Mt for 24 h was determined via scratch wound (n = 3) (**a**) and transwell (n = 3) (**b**) assays, and proliferation (n = 3) was determined by DAPI cell counting (**c**). Cell counting results were normalized to the NT group. The scale bars represent 500 µm. * *p* < 0.05; ** *p* < 0.01; **** *p* < 0.0001 vs. TGF-β group.

Fibroblast migration into wound areas represents one of the initial steps in tissue repair [72], but persistent migration can lead to excessive remodeling and expansion of fibrotic lesions [73]. Fibroblast migration is mediated by numerous pro-fibrotic factors, including TGF-β [74], with fibroblasts isolated from patients with pulmonary fibrosis displaying increased migratory potential compared to normal fibroblasts [75]. Herein, TGF-β1-stimulated fibroblasts underwent increased migration compared to naïve fibroblasts (Figure 7a,b). Notably, Dex-TPP/Mt treatment of fibroblasts stimulated with TGF-β1 significantly reduced cell migration in both scratch wound (Figure 7a) and transwell assays (Figure 7b). Similarly, fibroblast proliferation is a requisite in the early stages of wound healing, proving critical in the attempt to repair damage. However, fibroproliferative disorders such as IPF have as their hallmarks fibroblast foci, or regions of ongoing injury and repair comprised of actively proliferating fibroblasts/myofibroblasts [76]. TGF-β stimulation has been shown to result in increased fibroblast proliferation [77,78,79]. The metabolic reprogramming towards glycolysis described above is meant to enable fibroblasts to fulfill the energy demands that are required for protein synthesis and proliferation by switching to the more rapid mechanism of energy generation compared to OXPHOS [80]. Herein, Dex-TPP/Mt treatment of TGF-β1-stimulated fibroblasts resulted in a significant decrease in fibroblast proliferation compared to non-treated controls (Figure 7c).

Several attempts have been made to target TGF-β for the treatment of fibrosis, including the use of antibodies, peptides, and receptor decoys [81]. Unfortunately, these strategies have failed to impact fibrotic progression in patients [82]. Treatments have also aimed at decreasing fibrosis by targeting intracellular phosphorylation of Smad2/3 [83]. These include sequestration of Smad2/3 by Smad7 [84], as well as the Smad3 inhibitor SIS3, which has been shown to decrease ECM protein expression [85]. Studies have also examined targeting specific steps in glycolysis in fibroblasts in attempts to impact fibrotic progression. Nayak et al. explored the HIF-1α inhibitor YC-1 [3-(5′-hydroxymethyl-2′-furyl)-1-benzyl indazole] as a potential treatment for chronic kidney disease-induced fibrosis, highlighting a reduction in ECM accumulation and ROS production, among many other therapeutic effects [86]. Yin et al. demonstrated that the drug Lonidamine decreased TGF-β-stimulated fibroblast pro-fibrotic gene expression and migration [57]. Most recently, Gao et al. showed that disruption of the PKM2 tetramer with compound 3 k, an antagonist of PKM2, led to a reduction in the levels of p-Smad3, αSMA, Col1, and Fn [60]. Given the significance of fibroblast proliferation and migration in fibrosis, these processes also represent attractive targets for therapy. Zhao et al. demonstrated that activation of focal adhesive kinase (FAK) is required for fibroblast migration, and the FAK inhibitor PF-573228 inhibited fibroblast migration in a dose-dependent manner [73]. In another study, Manso et al. showed that the FAK inhibitor FAK-related nonkinase (FRNK) inhibited the platelet-derived growth factor-BB (PDGF-BB)-induced migration of cardiac fibroblasts [87]. With regards to proliferation, increased levels of 3′, 5′ cyclic adenosine monophosphate (cAMP) were found to be associated with cell processes in fibrosis, and agonists of the cAMP pathway inhibited fibroblast proliferation and differentiation [88]. 

Dex-TPP/Mt treatment affected several glycolytic enzymes and cell processes discussed above that have been targeted in previous studies by other groups. These findings are in line with a recent study by our group, wherein enhanced glycolytic activity in M1 macrophages was associated with a pro-inflammatory phenotype of these cells and that Dex-TPP/Mt treatment significantly reduced glycolytic enzyme upregulation and consequently, cytokine production [24]. Our findings demonstrate that enhanced OXPHOS and ATP production is associated with a lessened glycolytic phenotype and an abrogation of FMT and ECM deposition. It is important to note that mitochondria for this and prior studies [21,24] were isolated from liver, which proves thermodynamically efficient at ATP production, using OXPHOS in a manner that optimizes maximal ATP production while minimizing energy expenditure [89]. However, the liver does not represent the only source of mitochondria for purposes of transplantation, with mitochondria from other organs and tissues exhibiting high OXPHOS rates [90]. As an example, muscle tissue has a greater density of mitochondria specifically designed for enhanced OXPHOS capacity to quickly fulfill the demand for high ATP generation required by contracting muscles during physical activity [91]. Moreover, the OXPHOS capacity of skeletal muscle-derived mitochondria can be increased via exercise training [92,93]. Thus, mitochondria derived from muscle, in particular skeletal muscle, are just one example of an attractive source of mitochondria for future clinical applications of strategies involving mitochondrial transplantation.

To the best of our knowledge, this study represents the first report of mitochondrial transfer as a treatment strategy targeting pro-fibrotic programming in fibroblasts. Recent research efforts by us and others have shown the therapeutic potential of mitochondrial transfer in several diseases. Our laboratory recently showed that delivery of mitochondria to macrophages in atherosclerotic plaques led to reduced plaque area in a mouse model of atherosclerosis [24]. Xie et al. recently showed that mitochondrial dysfunction is associated with cerebral ischemia-reperfusion (I/R) injury and that delivery of exogenous mitochondria decreased ROS and apoptosis, increased cell viability, reduced infarct size, and ameliorated neurobehavioral deficits [94]. Recently, Lin et al. demonstrated that mitochondrial dysfunction is a hallmark of neuroinflammatory responses and neuronal death in spinal cord injury (SCI) and that mitochondrial transplantation to injured spinal cords resulted in improved recovery of locomotor and sensory functions and alleviated SCI-induced cell apoptosis and inflammatory responses [95]. Taken together, mitochondrial transfer can impact a variety of diseases whose hallmarks include mitochondrial remodeling and dysfunction.

## 3. Materials and Methods

### 3.1. Materials

Dextran (Dex, Mw 150 kDa) was purchased from Sigma-Aldrich (St. Louis, MO, USA), and (5-Carboxypentyl) triphenylphosphonium bromide (TPP-COOH) obtained from Alfa Aesar (Lancashire, UK). Fluorescein isothiocyanate, isomer 1, 95% (FITC, *λ*_ex_ = 496 nm and *λ*_em_ = 519 nm) was purchased from Thermo Fisher Scientific (Waltham, MA, USA). MRC-5 human lung fibroblast cells were purchased from the American Type Culture Collection (ATCC, Manassas, VA, USA) and cultured in Eagle’s Minimum Essential Medium (EMEM) with 2 mM L-Glutamine, 1 mM sodium pyruvate, and 1500 mg/L sodium bicarbonate supplemented with 1% Penstrep (Corning Inc., Corning, NY, USA) and 10% FBS (Corning Inc.) at 37 °C with 5% CO_2_. Recombinant Human TGF-β1 (7754-BH-025/CF) was purchased from R&D Systems (Minneapolis, MN, USA). All chemicals and reagents, unless otherwise specified, were purchased from Sigma-Aldrich.

### 3.2. Animals

All animal studies were approved by the Institutional Animal Care and Use Committee of the Houston Methodist Research Institute. C57BL/6J male mice (8 weeks) were obtained from Jackson Laboratory (Bar Harbor, ME, USA).

### 3.3. Dex-TPP Synthesis, Characterization, and Functionalization of Isolated Mitochondria

Dex was conjugated to TPP as previously published [21], and conjugation was confirmed by ^1^H NMR using a Varian 400 MHz NMR spectrometer (Santa Clara, CA, USA) as previously reported. For cell uptake, FITC was conjugated to Dex-TPP at a molar ratio of 1:10 Dex-TPP:FITC. Mitochondria were isolated from livers harvested from euthanized C57BL/6J mice based on a previously published procedure [21]. Liver mitochondria were used in this study due to their ATP production efficiency [89] and increased isolation yields compared to other tissues [96]. Dex-TPP was mixed with mitochondria (Dex-TPP/Mt) at a weight ratio of 1.9:1 Dex-TPP:mitochondria protein. In all experiments involving Dex-TPP/Mt treatment, Dex-TPP/Mt at a concentration of 10 μg of mitochondrial protein per 1.5 × 10^4^ cells was added to cells simultaneously with TGF-β1.

### 3.4. Fluorescence Microscopy Examination of Fibronectin and α-Smooth Muscle Actin Expression

Fibronectin (FN) and α-smooth muscle actin (αSMA) expression were examined using confocal microscopy. MRC-5 cells were seeded into 8-chamber glass slides (1 × 10^4^ cells/well) and incubated with different TGF-β1 concentrations (2, 5, and 10 ng/mL) or with TGF-β1 (5 ng/mL) and FITC-conjugated Dex-TPP/Mt for 24 h. Cells were washed with 1× PBS (Corning Inc.) three times and fixed with 4% paraformaldehyde (PFA) (Thermo Fisher Scientific). Following a second wash with 1× PBS, cells were permeabilized with 0.1% Triton (Thermo Fisher Scientific) in 1× PBS for 1 h and washed with 1× PBS twice. Proteins were blocked with 2% bovine serum albumin (BSA, GoldBio, St. Louis, MO, USA) in 1× PBS at RT for 1 h, followed by incubation with primary antibodies overnight at 4 °C. The following primary antibodies were used: fibronectin (ab2413, Abcam, Cambridge, UK) and αSMA (ab124964, Abcam). Cells were washed with 1× PBS twice and incubated with goat anti-rabbit IgG-CFL 647 (sc-362292, Santa Cruz Biotechnology, Inc., Dallas, TX, USA) at RT for 2 h. Cells were washed with 1× PBS twice, and the cytoskeleton was stained with Acti-Stain 555 Phalloidin (PHDH1-A, Cytoskeleton, Inc., Denver, CO, USA) for 1 h. Cells were then washed with 1× PBS twice, and nuclei were labeled with DAPI (4′,6-diamidino-2-phenylindole, Thermo Fisher Scientific) for 20 min. Confocal microscopy images were captured using a Nikon A1 Confocal Imaging System (Nikon, Tokyo, Japan). 

### 3.5. Flow Cytometry and Confocal Microscopy for Uptake Examination

MRC-5 cells were seeded into 6-well plates (2.0 × 10^5^ cells/well) and incubated with TGF-β1 (5 ng/mL) and Dex-TPP/Mt labeled with FITC for 24 h. Cells were washed, detached, and, following centrifugation, resuspended with BD Cytofix/Cytoperm (BD Biosciences, Franklin Lakes, NJ, USA). Uptake was analyzed using an LSRII Flow Cytometer (BD Biosciences). Dex-TPP/Mt uptake was corroborated via confocal microscopy, with images captured using a Nikon A1 Confocal Imaging System (Nikon). MRC-5 cells were seeded and stained with Acti-Stain 555 Phalloidin and DAPI as previously described. 

### 3.6. Western Blot

MRC-5 cells (1.5 × 10^5^ cells/well) were seeded into 6-well plates. Cells were initially starved for 4 h with 2% FBS medium and were then treated with different TGF-β1 concentrations (2, 5, and 10 ng/mL) or with TGF-β1 (5 ng/mL) and Dex-TPP/Mt for 24 h. Subsequently, cells were washed with 1× PBS and lysed with RIPA Cell Lysis Buffer with EDTA (GenDEPOT, Houston, TX, USA) containing 1× Protease/Phosphatase Inhibitor Cocktail (100×, Cell Signaling Technology, Danvers, MA, USA). Protein samples were obtained through centrifugation, and quantification was performed with a BCA protein assay kit (Bio-Rad, Hercules, CA, USA). After electrophoresis on Mini-PROTEAN TGX precast gels (Bio-Rad), proteins were transferred to a nitrocellulose blotting membrane (Bio-Rad). The membrane was blocked with 5% BSA in tris buffered saline with 0.1% tween 20 (GenDEPOT), followed by incubation with primary antibodies overnight at 4 °C. The following primary antibodies were used: fibronectin (ab2413, Abcam), collagen type I (ab34710, Abcam), phospho-Smad2 (Ser465/467)/Smad3 (Ser423/425) (8828, Cell Signaling Technology), Smad2/3 (8685, Cell Signaling Technology), α-Smooth Muscle Actin (14968, Cell Signaling Technology), GAPDH (5174, Cell Signaling Technology), HIF-1α (14179, Cell Signaling Technology), Anti-Lactate Dehydrogenase B/LDH-B (ab240482, Abcam), hexokinase II (HK2) (2867, Cell Signaling Technology), and PKM2 (4053, Cell Signaling Technology). Membranes were incubated with HRP-conjugated secondary antibodies (Cell Signaling Technology) at RT for 2 h. Protein bands were detected using Immobilon Western HRP substrate (MilliporeSigma, Burlington, MA, USA) and visualized using a ChemiDoc-XRS imaging system (Bio-Rad).

### 3.7. MTT Assay 

MRC-5 cells (1.0 × 10^4^ cells/well) were seeded into 96-well plates. Cells were initially starved for 4 h with 2% FBS medium and then treated with different TGF-β1 concentrations (2, 5, and 10 ng/mL) for 24 h. Subsequently, cells were washed with 1× PBS twice, and 100 µL of 3-(4,5-Dimethylthiazol-2-yl)-2,5-diphenyltetrazolium bromide (MTT) dissolved in dimethyl sulfoxide (DMSO, 10 mg/mL) diluted in serum-free media 1:10 (*v*/*v*) was added in each well, and the plate was incubated at 37 °C for 2 h. The medium was then removed, 100 µL of DMSO was added to each well, and the plate was incubated for 30 min at RT under agitation. Absorbance was measured at 570 nm with a FLUOstar Omega microplate (BMG Labtech, Berlin, Germany). Cell viability (%) was calculated using the following formula:

$$\frac{A_{sample}}{A_{control}}\times 100.$$

### 3.8. Extracellular Flux Analysis

MRC-5 cells (4 × 10^3^ cells/well) were seeded in a Seahorse XFe96 cell culture microplate (Agilent Technologies, Santa Clara, CA, USA) and treated with different TGF-β1 concentrations (2, 5, and 10 ng/mL) or with TGF-β1 (5 ng/mL) and Dex-TPP/Mt for 24 h. OCR and ECAR were measured using the Seahorse XFe96 Analyzer (Agilent, Santa Clara, CA, USA), as recommended by the manufacturer for the Seahorse XF Cell Mito Stress Test Kit (Agilent Technologies). OCR and ECAR measurements were normalized to cell number by nuclei staining with DAPI and counted in a blind fashion using ImageJ software version 1.53 (NIH, Bethesda, MD, USA). The entire area of the well was captured using a Keyence BZ-X810 microscope (Keyence, Itasca, IL, USA).

### 3.9. ATP and Glucose Consumption Assays

MRC-5 cells (8.0 × 10^3^ cells/well) were seeded into 96-well plates and treated with different TGF-β1 concentrations (2, 5, and 10 ng/mL) or with TGF-β1 (5 ng/mL) and Dex-TPP/Mt for 24 h. ATP was measured using the ATPlite Luminescence Assay System (PerkinElmer, Waltham, MA, USA) according to the manufacturer’s protocol. Glucose consumption was measured using the Glucose Colorimetric/Fluorometric Assay Kit (BioVision Incorporated, San Francisco, CA, USA) according to the manufacturer’s protocol. Luminescence and fluorescence were measured using a Spark multimode microplate reader (Tecan, Männedorf, Switzerland).

### 3.10. ROS Examination

MRC-5 cells were seeded into 4-chamber glass slides (2 × 10^4^ cells/well) and incubated with TGF-β1 (5 ng/mL) or with TGF-β1 (5 ng/mL) and Dex-TPP/Mt for 24 h. Cells were washed twice with 1× PBS and incubated with 200 µL of MitoSOX™ Mitochondrial Superoxide (Thermo Fisher Scientific) diluted in serum-free media 1:1000 (*v*/*v*) for 20 min at 37 °C. Cells were washed with 1× PBS twice and fixed with 4% paraformaldehyde (PFA) (Thermo Fisher Scientific). Cells were then washed with 1× PBS twice, and nuclei were labeled with DAPI for 20 min. Confocal microscopy images were captured using a Nikon A1 Confocal Imaging System (Nikon). ROS quantification was analyzed using an LSRII Flow Cytometer (BD Biosciences) as previously described.

### 3.11. Cell Proliferation Evaluation

MRC-5 cells (1 × 10^3^ cells/well) were seeded into 96-well plates and incubated with different TGF-β1 concentrations (2, 5, and 10 ng/mL) or with TGF-β1 (5 ng/mL) and Dex-TPP/Mt for 24 h. Cells were washed with 1× PBS and fixed with 4% PFA. Cell nuclei were labeled with DAPI. The entire area of the well was captured using a Keyence BZ-X810 microscope. Cell numbers were counted in a blind fashion using ImageJ software.

### 3.12. Cell Migration Studies

MRC-5 cell migration was examined via wound healing and transwell assays. For the wound healing assay, MRC-5 cells (2.5 × 10^5^ cells/well) were seeded into 6-well plates. After 24 h, the wound was created with a 1 mL pipette tip, and the cells were washed with 1× PBS twice and treated with TGF-β1 (5 ng/mL) or with TGF-β1 (5 ng/mL) and Dex-TPP/Mt in complete medium (10% FBS EMEM). The wound was evaluated by obtaining images at the following timepoints: 0 and 24 h using an EVOS Cell Imaging System (Thermo Fisher Scientific). The wound area was calculated as the average of four images for each timepoint using ImageJ software. For the transwell assay, MRC-5 cells (4.0 × 10^4^ cells/well) were seeded into 24-well plates in the upper compartment of an 8 μm chamber (Greiner Bio-One, Kremsmünster, AT, USA) with 0.5% FBS EMEM and treated with Dex-TPP/Mt. In the lower chamber, 0.5% FBS EMEM medium with TGF-β1 (5 ng/mL) was added. After incubation for 24 h, the chambers were removed, and cells were fixed with 4% PFA. Cells were then washed with 1× PBS twice and stained with 0.1% crystal violet (Fisher Scientific International, Inc., Pittsburgh, PA, USA) for 30 min. Non-migrated cells were removed with a cotton swab. Migrated cells were observed with a Keyence BZ-X810 microscope. Finally, cells were eluted with 90% acetic acid (Sigma-Aldrich), and the absorbance was measured at 590 nm with a FLUOstar Omega microplate (BMG Labtech, Berlin, Germany).

### 3.13. Statistical Analysis

All data represent the mean ± standard error of the mean (SEM). Data were evaluated with GraphPad Prism 9.5.1 (GraphPad Software, San Diego, CA, USA) using unpaired ordinary one-way ANOVA followed by Dunnett’s multiple comparison tests. Unless otherwise stated, values represent the mean ± SEM. * *p* < 0.05, ** *p* < 0.01, *** *p* < 0.001, and **** *p* < 0.0001 were considered statistically significant.

## 4. Conclusions

Our findings highlight the potential of mitochondrial delivery to fibroblasts as a treatment for fibrosis. Results from this study show that TGF-β stimulation of fibroblasts results in a pro-fibrotic program that is associated with impaired cell bioenergetics in the form of increased glycolysis and mitochondrial dysfunction. Mitochondrial transfer to TGF-β-stimulated fibroblasts induced metabolic alterations in these cells that hindered a pro-fibrogenic phenotype. Future work will involve the exploration of Dex-TPP/Mt as a therapeutic strategy in a variety of diseases characterized by fibrosis, as well as diseases whose hallmarks include mitochondrial dysfunction. This work demonstrates that mitochondrial replenishment represents a powerful treatment modality due to its ability to modulate aberrant bioenergetics that impact processes that ultimately drive fibrotic progression.

## Data Availability

The data presented in this study are available on request from the corresponding author.

## Supplementary Materials

The supporting information can be downloaded at: https://www.mdpi.com/article/10.3390/ijms241310913/s1.

## Abbreviations

ATCC	American Type Culture Collection
BMDM	Bone marrow-derived macrophage
cAMP	3′, 5′ cyclic adenosine monophosphate
CMs	Cardiomyocytes
Col1	Type 1 collagen
DAPI	4′,6-diamidino-2-phenylindole
Dex	Dextran
Dex-TPP/Mt	Dextran and triphenylphosphonium mitochondria
DMSO	Dimethyl sulfoxide
ECAR	Extracellular acidification rate
ECM	Extracellular matrix
EMEM	Eagle’s Minimum Essential Medium
ETC	Electron transport chain
FAK	Focal adhesive kinase
FGF-2	Fibroblast growth factor 2
FITC	Fluorescein isothiocyanate
FMT	Fibroblast-to-myofibroblast transition
FN	Fibronectin
FRNK	FAK-related nonkinase
HF	High-fat
HIF-1α	Hypoxia-inducible factor-1α
HK2	Hexokinase-2
hVFF	Human vocal fold fibroblasts
I/R	Ischemia-reperfusion
IPF	Idiopathic pulmonary fibrosis
LDH	Lactate dehydrogenase
mtROS	Mitochondrial reactive oxygen species
MTT	3-(4,5-Dimethylthiazol-2-yl)-2,5-diphenyltetrazolium bromide
NAFLD	Nonalcoholic fatty liver disease
NASH	Nonalcoholic steatohepatitis
NOX4	NADPH oxidase 4
NT	Non-treated
OCR	Oxygen consumption rate
OXPHOS	Oxidative phosphorylation
PDGF-BB	Platelet-derived growth factor-BB
PEP	Phosphoenolpyruvate
PFA	Paraformaldehyde
PGDM	Pregestational diabetes mellitus
PHD2	Prolyl hydroxylase domain-containing protein 2
PHDH1-A	Acti-Stain 555 Phalloidin
PKM2	Pyruvate kinase isozyme M2
ROS	Reactive oxygen species
SCI	Spinal cord injury
SEM	Standard error of the mean
TGF-β	Transforming growth factor-β
TNBC	Triple negative breast cancer
TPP	Triphenylphosphonium
TPP-COOH	Triphenylphosphonium bromide
VEGF	Vascular endothelial growth factor
αSMA	α-smooth muscle actin
