# Supplementary material for: Polymer-Functionalized Mitochondrial Transplantation to Fibroblasts Counteracts a Pro-Fibrotic Phenotype
Source: Int J Mol Sci. 2023 Jun 30;24(13):10913. doi: 10.3390/ijms241310913 (PMC10342003; doi:10.3390/ijms241310913)
Supplement: Supplementary file 1 [file ijms-24-10913-s001.zip › ijms-2410186-supplementary.pdf]

Supplementary Material

Polymer-Functionalized Mitochondrial Transplantation to Fibroblasts Counteracts a Pro-Fibrotic Phenotype

Gherardo Baudo, Suhong Wu, Matteo Massaro, Haoran Liu, Hyunho Lee, Aijun Zhang, Dale J. Hamilton, and Elvin Blanco

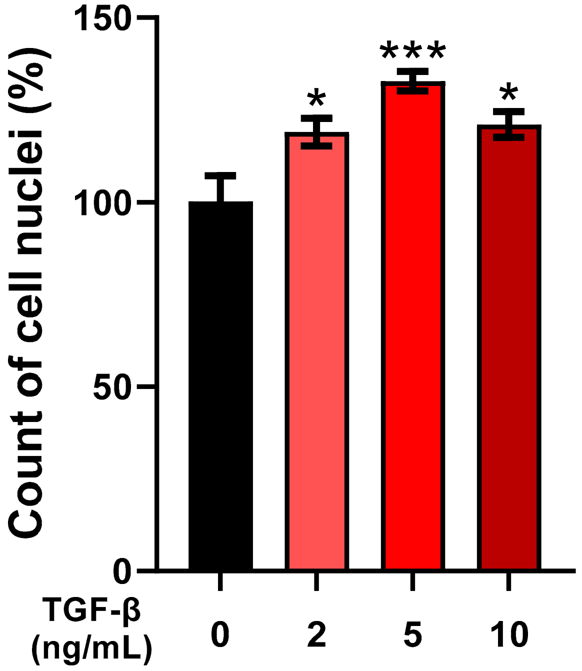

Figure S1. Effect of TGF-β on fibroblast proliferation. Fibroblast proliferation 24 h after TGF-β treatment as determined by DAPI cell counting (n = 3). Results were normalized to the 0 ng/mL TGF-β group. *$p < 0.05$; ***$p < 0.001$ vs 0 ng/mL TGF-β group.

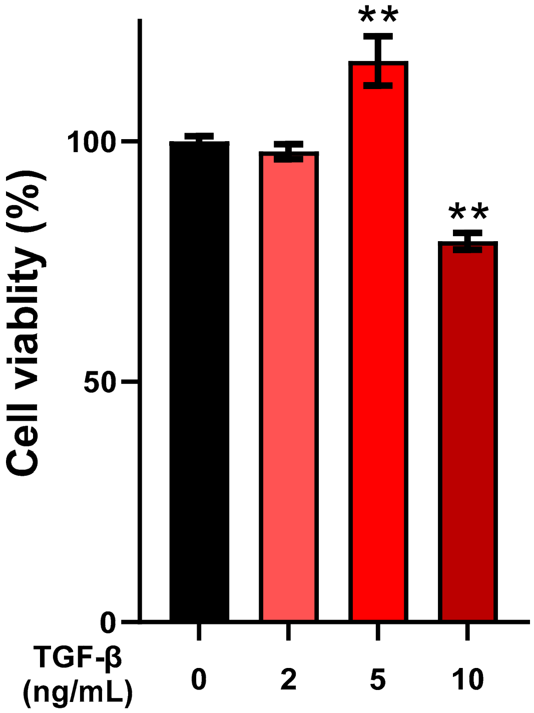

Figure S2. Effect of increasing concentrations of TGF-β on fibroblast viability. Fibroblast viability 24 h after TGF-β treatment as determined by MTT assay (n = 3). Results were normalized to the 0 ng/mL TGF-β group. **$p < 0.01$ vs 0 ng/mL TGF-β group.

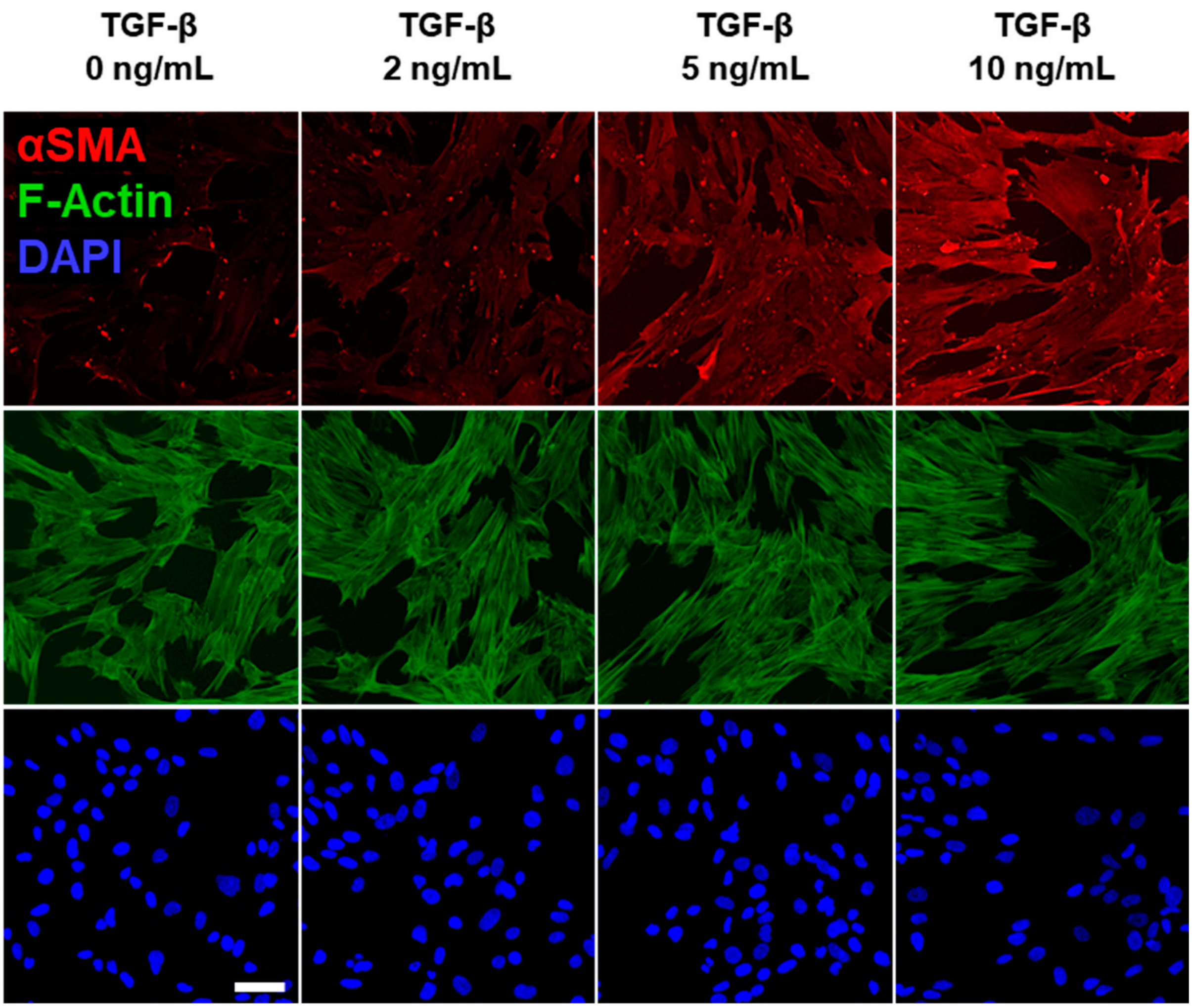

Figure S3. Effect of TGF-β on the expression of the myofibroblast marker α-SMA. Representative immunofluorescence micrographs highlighting expression of αSMA in TGF-β-stimulated fibroblasts. αSMA appears red, F-actin is represented in green, and nuclei are highlighted by DAPI staining. The scale bar represents 100 μm.

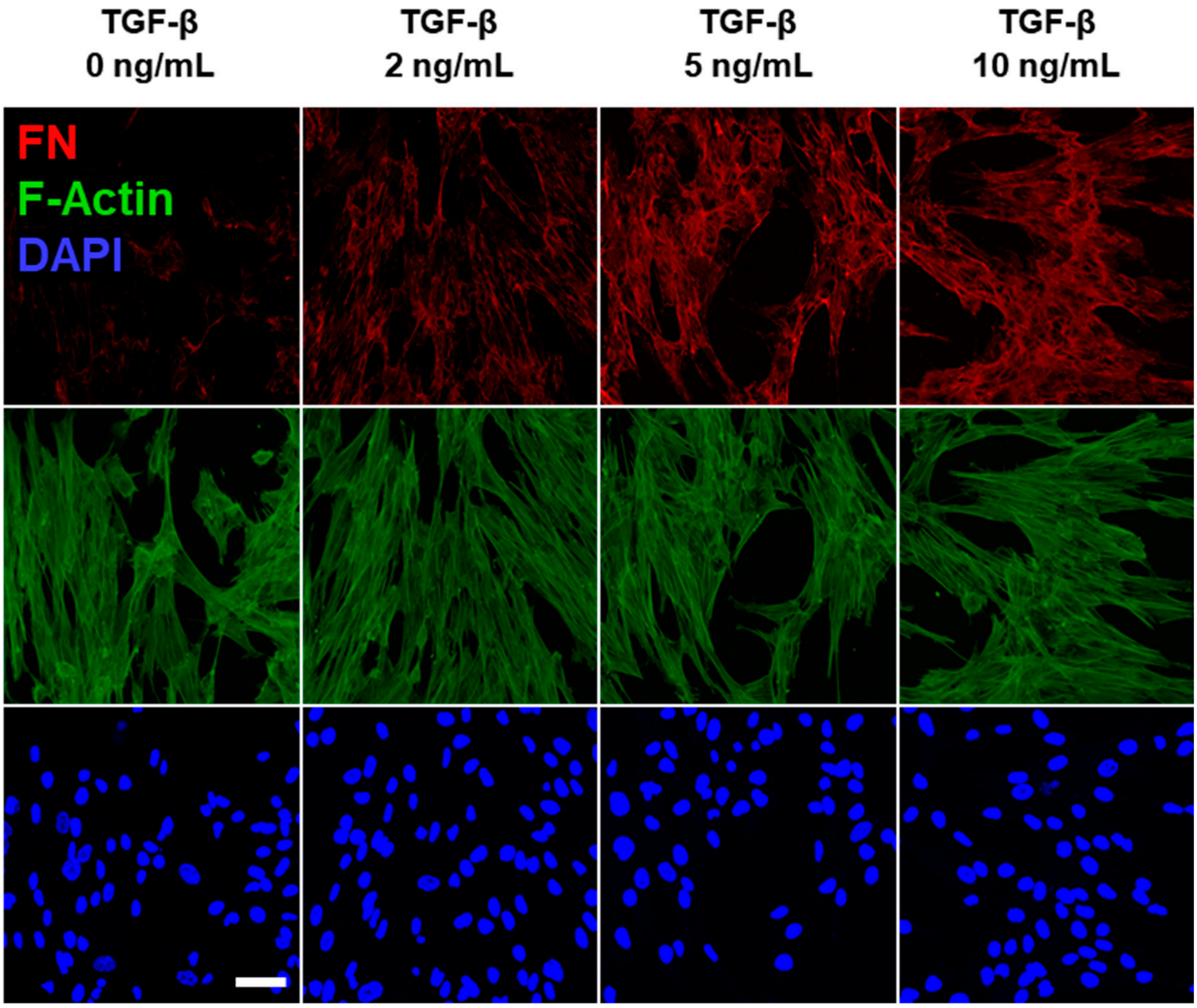

Figure S4. Effect of TGF-β on the expression of the extracellular matrix protein fibronectin (FN). Representative immunofluorescence micrographs highlighting expression of FN in TGF-β-stimulated fibroblasts. FN appears red, F-actin is represented in green, and nuclei are highlighted by DAPI staining. The scale bar represents 100 μm.

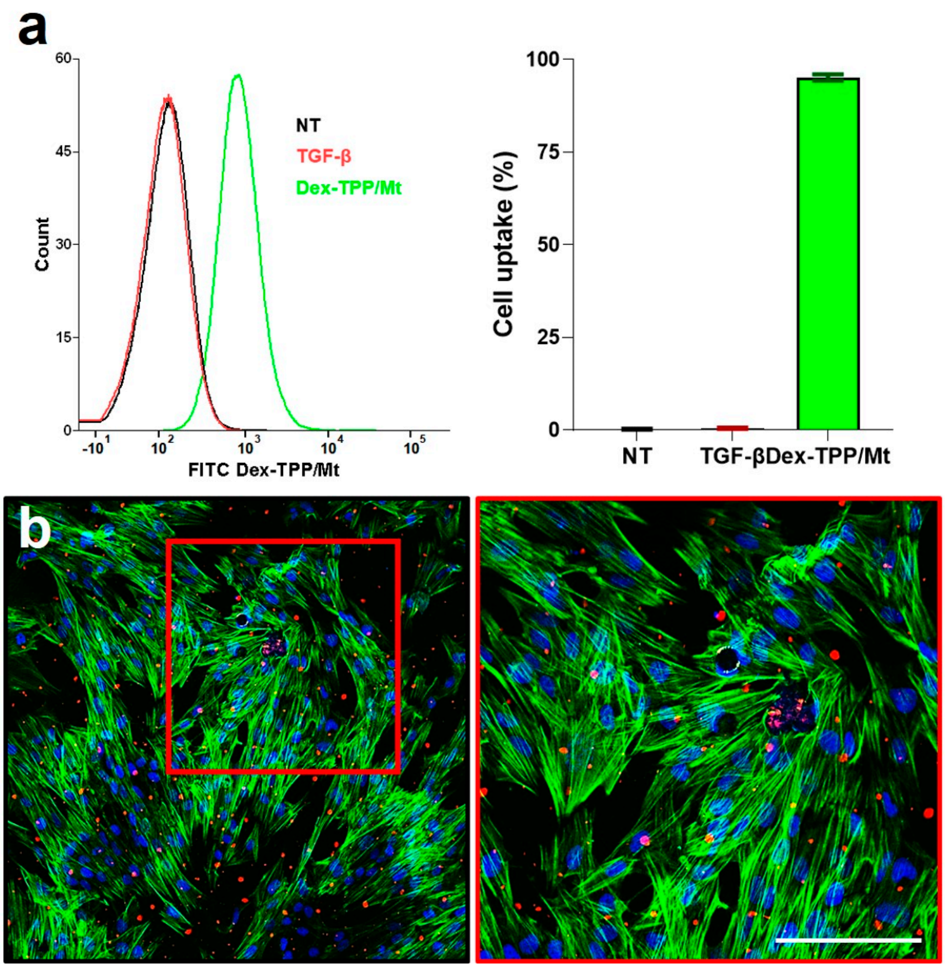

Figure S5. Dex-TPP/Mt uptake in TGF-β-stimulated fibroblasts. **a**) Flow cytometry histogram and percentage of TGF-β-stimulated fibroblasts with FITC-labeled Dex-TPP/Mt-associated fluorescence at a timepoint of 24 h (n = 3). **b**) Representative confocal microscopy images of TGF-β-stimulated fibroblasts treated with FITC-labeled Dex-TPP/Mt for 24 h. FITC-labeled Dex-TPP/Mt appear as red, F-actin is represented by green, and DAPI-stained nuclei appear as blue. The scale bar represents 100 μm.

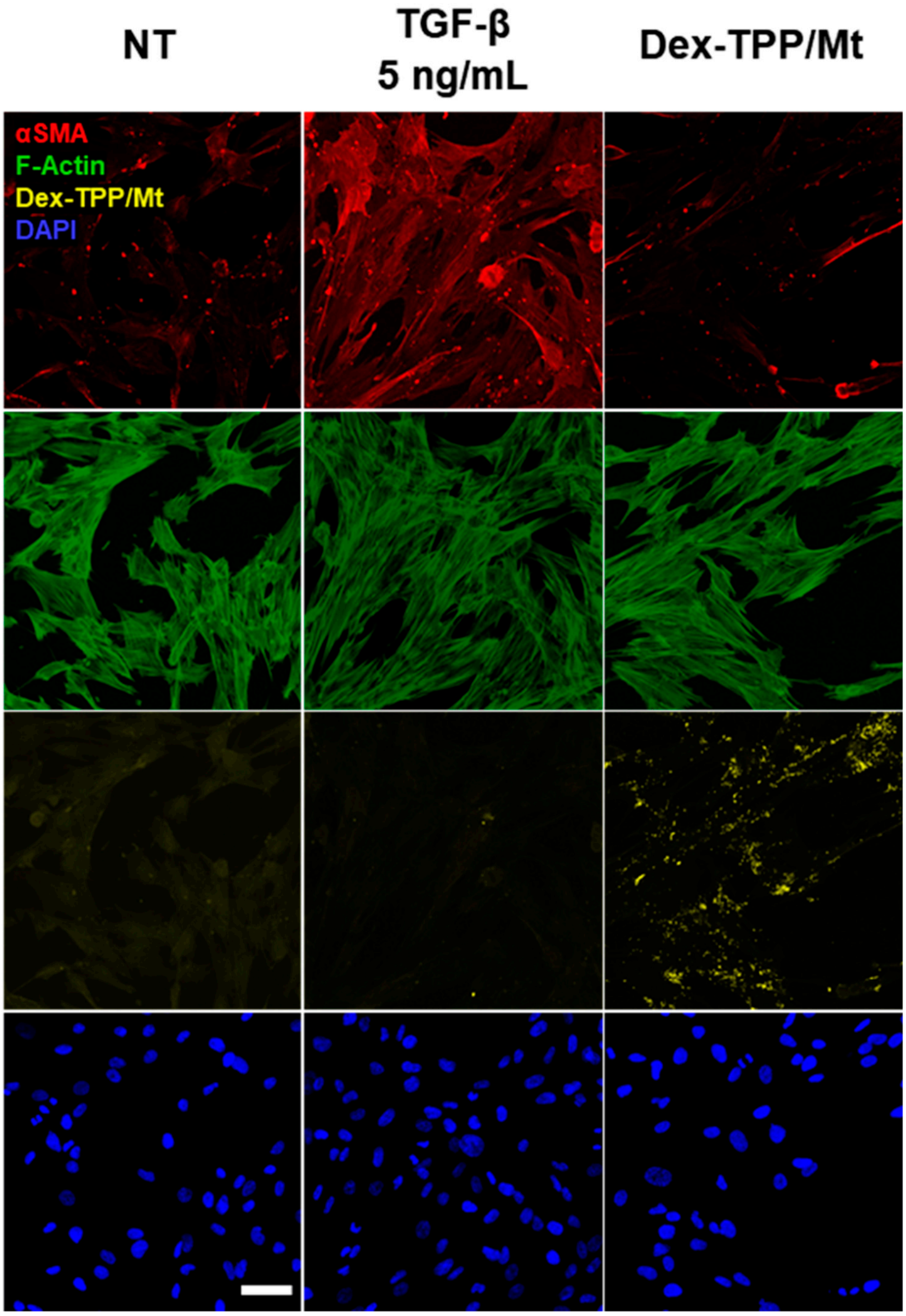

Figure S6. Effect of Dex-TPP/Mt treatment on the expression of the myofibroblast marker αSMA. Representative immunofluorescence micrographs highlighting expression of αSMA in non-treated fibroblasts (NT), TGF-β-stimulated fibroblasts (TGF-β), and TGF-β-stimulated fibroblasts treated with FITC-labeled Dex-TPP/Mt for 24 h. αSMA appears red, F-actin is represented in green, FITC-labeled Dex-TPP/Mt appear yellow, and nuclei are highlighted by DAPI staining. The scale bar represents 100 μm.

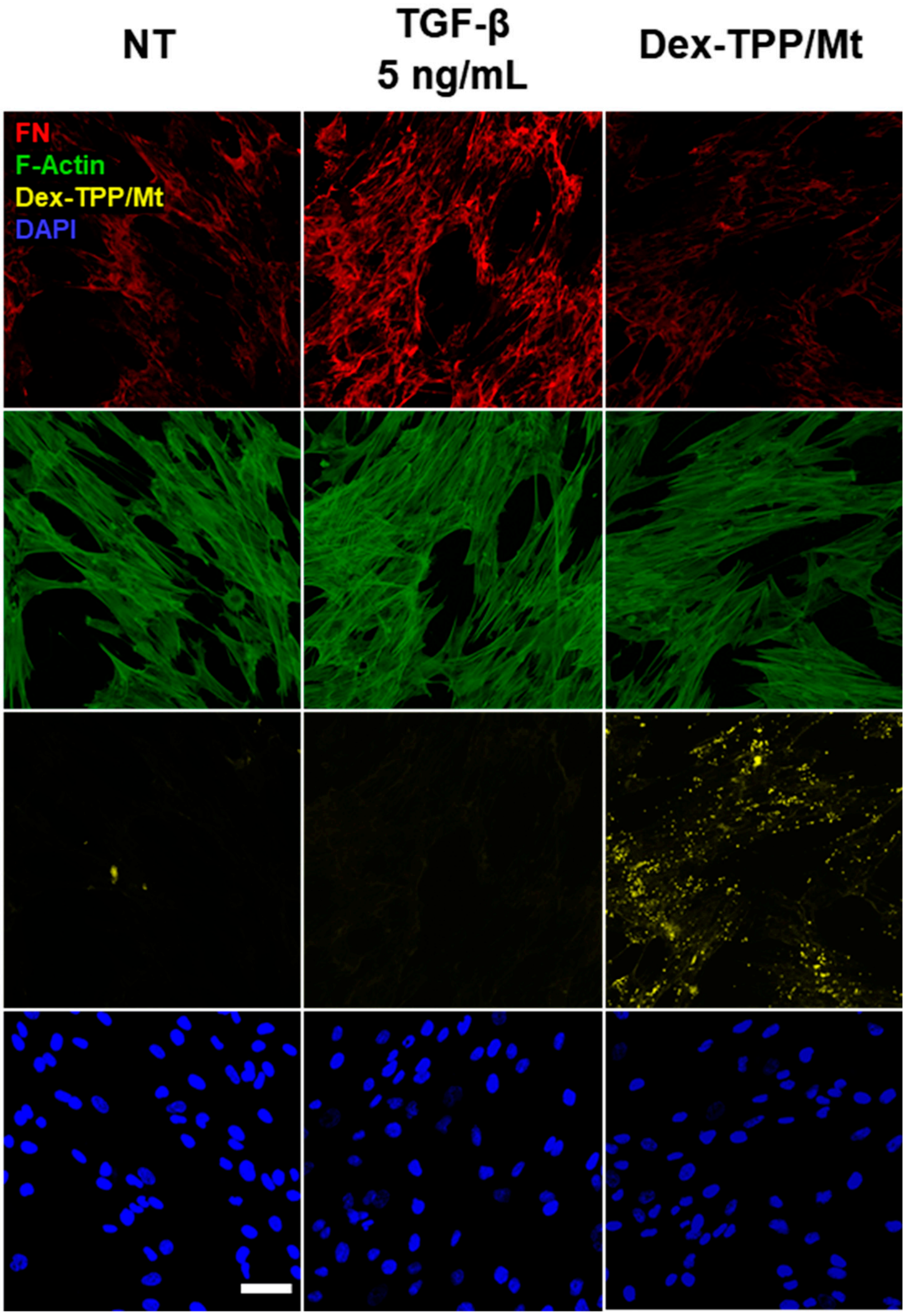

Figure S7. Effect of Dex-TPP/Mt treatment on the expression of the extracellular matrix protein fibronectin (FN). Representative immunofluorescence micrographs highlighting expression of αSMA in non-treated fibroblasts (NT), TGF-β-stimulated fibroblasts (TGF-β), and TGF-β-stimulated fibroblasts treated with FITC-labeled Dex-TPP/Mt for 24 h. FN appears red, F-actin is represented in green, FITC-labeled Dex-TPP/Mt appear yellow, and nuclei are highlighted by DAPI staining. The scale bar represents 100 μm.
